# Metabolomic and Transcriptomic Analyses of *Escherichia coli* for Efficient Fermentation of L-Fucose

**DOI:** 10.3390/md17020082

**Published:** 2019-01-29

**Authors:** Jungyeon Kim, Yu Eun Cheong, Inho Jung, Kyoung Heon Kim

**Affiliations:** 1 Department of Biotechnology, Graduate School, Korea University, Seoul 02841, Korea; kim131812@korea.ac.kr (J.K.); yecheong0127@korea.ac.kr (Y.E.C.); wind9887@hanmail.net (I.J.); 2 Forest Product Analysis and Certification Division, Korea Forestry Promotion Institute, Seoul 07570, Korea

**Keywords:** fucose, fermentation, metabolomics, transcriptomics, *Escherichia coli*, brown macroalgae

## Abstract

L-Fucose, one of the major monomeric sugars in brown algae, possesses high potential for use in the large-scale production of bio-based products. Although fucose catabolic pathways have been enzymatically evaluated, the effects of fucose as a carbon source on intracellular metabolism in industrial microorganisms such as *Escherichia coli* are still not identified. To elucidate the effects of fucose on cellular metabolism and to find clues for efficient conversion of fucose into bio-based products, comparative metabolomic and transcriptomic analyses were performed on *E. coli* on L-fucose and on D-glucose as a control. When fucose was the carbon source for *E. coli*, integration of the two omics analyses revealed that excess gluconeogenesis and quorum sensing led to severe depletion of ATP, resulting in accumulation and export of fucose extracellularly. Therefore, metabolic engineering and optimization are needed for *E. coil* to more efficiently ferment fucose. This is the first multi-omics study investigating the effects of fucose on cellular metabolism in *E. coli*. These omics data and their biological interpretation could be used to assist metabolic engineering of *E. coli* producing bio-based products using fucose-containing brown macroalgae.

## 1. Introduction

Owing to increasing concerns surrounding energy security and global warming, not only lignocellulose [1,2,3] but also macroalgae are considered as important biomass resources for the production of biofuels and bio-based products using microorganisms [4]. Macroalgae are widely used owing to their many advantages over terrestrial plants, since they do not require arable land or fertilizers during cultivation, and have high carbohydrate but low lignin content [4,5,6]. In macroalgae, alginate, laminarin, mannitol, and fucoidan are the major carbohydrates [7,8]. To convert these carbohydrates in brown macroalgae into biofuels and chemicals in a more efficient and economical manner, understanding the metabolism of the monomeric sugars of these carbohydrates in fermentative microorganisms is essential [1,2,3,8].

L-Fucose is the main monomeric sugar of fucoidan in brown macroalgae [8,9,10]. Although the current price of L-fucose is high [11], it has high potential to be used for production of bio-based products owing to its high abundance in diverse environments such as brown macroalgae and biofilms [10,11,12]. In addition, L-fucose is used as a substrate for the microbial production of 2-fucosyllactose and 3-fucosyllactose [13,14,15]. With such microbial conversion of fucose into bio-based products, it is important to understand the fermentation of fucose and its related cellular metabolism in industrial microbial hosts such as *Escherichia coli* [8,10,16]. Until now, metabolic enzymatic studies focusing on the kinase-dependent metabolic pathway involving the conversion of L-fucose into L-fuculose, L-fuculose-1-phosphate, and dihydroxyacetone phosphate (DHAP), have been performed using *E. coli* [17,18,19]. However, there are no studies regarding the intracellular metabolism of *E. coli* utilizing L-fucose, which could provide the design principles of microbial engineering for more efficient fermentation and conversion of fucose into bio-based products. Until now, only two microorganisms, *Lactobacillus rhamnosus* GG and *Sulfolobus solfataricus*, have been cultured on fucose for comparative transcriptomic, proteomic, and metabolomics analyses [20,21]. However, since these microorganisms are not industrial fermentative hosts, such analyses need to be performed on industrial strains such as *E. coli* [22].

Omics technologies, such as transcriptomics, proteomics, and metabolomics, can be effectively used in combination in order to study cellular metabolism [20,21,23,24,25]. In particular, metabolomics can identify global changes in metabolites which are directly related to cellular phenotypes [26]. Furthermore, to understand metabolite profile changes at the molecular level, it is necessary to combine metabolomics with transcriptomics, which reveals gene expression levels [20,21,23,24,25]. This is because changes in metabolite profiles can result from either up-regulation or down-regulation of gene expression [27]. Eventually, this multi-omics approach, combining metabolomics and transcriptomics, could indicate metabolic bottlenecks and metabolic flows [20,21,23,24,25].

In this study, the effects of L-fucose fermentation on the global metabolic network of *E. coli* K12 MG1655, one of the representative microorganisms in industrial production of various chemicals [16], was elucidated using metabolomic and transcriptomic analyses, and for more efficient conversion of L-fucose into bio-based products, possible targets of metabolic engineering were revealed. For metabolome analysis, gas chromatography/time of flight-mass spectrometry (GC/TOF–MS) and ultra-high performance liquid chromatography (UHPLC)/Q–Exactive mass spectrometry (MS) were used. For transcriptome analysis, a HiSeq 2000 sequencing system was used.

## 2. Results

### 2.1. Comparison of Growth and Fermentation Product Profiles of E. coli on Fucose and Glucose

**Figure 1:**
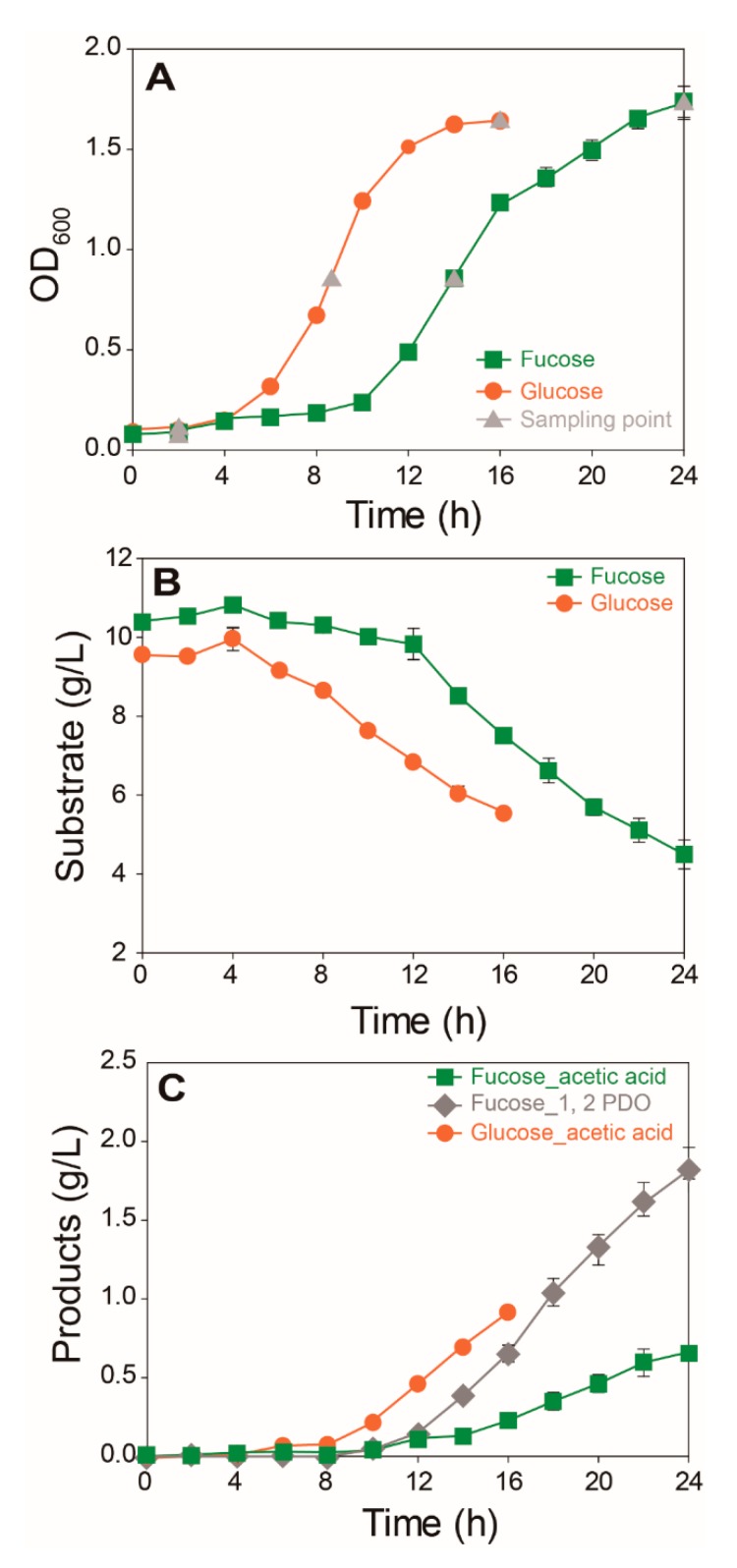
Comparison of the growth and fermentation product profiles of *E. coli* cultured on fucose and glucose as the carbon source (mean ± SD): (**A**) cell density recorded as optical density at 600 nm (OD_600_); (**B**) concentrations of substrate; and (**C**) by-products (three independent replicates).

Growth, substrate consumption, and production of main fermentation products were compared for *E. coli* cultured in M9 media containing fucose or glucose (Figure 1). *E. coli* cells cultured in fucose media showed notably lower growth rates (µ = 0.121 h^−1^) than those cultured in glucose media (μ = 0.223 h^−1^) (Figure 1A). Consumption of fucose and glucose (Figure 1B) and production of acetic acid and 1,2-propanediol (1,2-PDO) were compared (Figure 1C). *E. coli* cultured in fucose media produced two main fermentation products, namely acetic acid (0.66 g/L) and 1,2-PDO (1.82 g/L), after 24 h. In contrast, *E. coli* cultured in glucose media produced acetic acid (0.91 g/L) as the main fermentation product after 16 h.

### 2.2. Identification of Metabolites and Transcripts

In this study, 102 intracellular metabolites (Table S1A) and 53 extracellular metabolites (Table S1B) were identified using GC/TOF–MS. Eight intracellular cofactors, acetyl-CoA, AMP, ADP, ATP, NAD+, NADH, NADP+, and NADPH, were identified by UHPLC and Q-Exactive MS (Table S1C). A total of 4414 transcripts were identified using the HiSeq 2000 platform (Table S2).

### 2.3. Comparison of Intracellular Metabolite Profiles of E. coli on Fucose and Glucose

**Figure 2:**
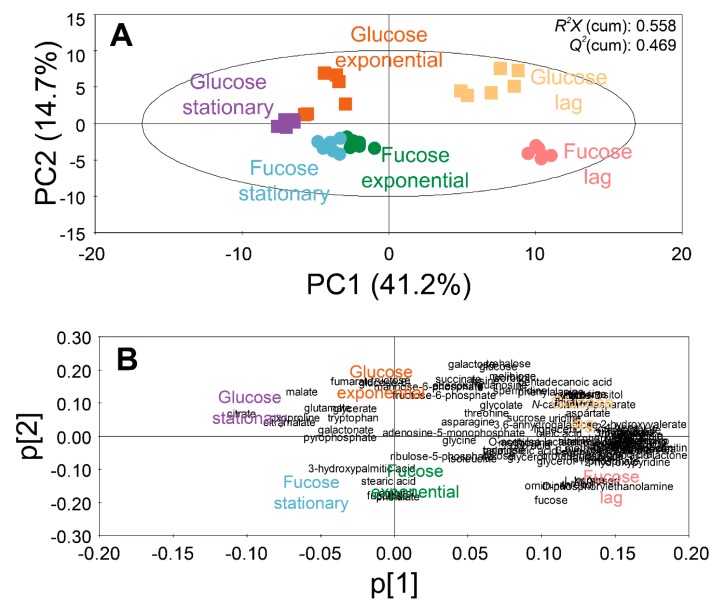
PCA (**A**) score and (**B**) loading plots of 102 intracellular metabolites of *E. coli* in the lag, exponential, and stationary phases cultured on fucose and glucose as the carbon source (six replicates; three independent replicates × two technical replicates).

To investigate the effect of fucose fermentation on intracellular metabolism, 102 intracellular metabolites of *E. coli* cultured in fucose and glucose media were compared. To compare the intracellular metabolite profiles between the two carbon sources (fucose and glucose) at three different growth phases (lag, exponential, and stationary phases), multivariate statistical analysis, principal component analysis (PCA), was performed (Figure 2).

A score plot of the PCA model using 102 intracellular metabolites showed the discrimination of metabolite profiles depending on different sugars and growth phases (Figure 2A). The quality of the PCA model with two components was represented by *R*^2^*X* (cumulative) of 0.558 and *Q*^2^ (cumulative) of 0.469. The high values of *R^2^X* and *Q^2^* indicate goodness of the fit, and predictive capability of the PCA model, respectively. The loading plot of the PCA model with the two principal components showed the contribution of each metabolite to the PCs (Figure 2B). The loading values of metabolites in the PCA models are listed in Table S3.

In the PCA model, the intracellular metabolite profiles of *E. coli* were clearly discriminated, based on growth phases and carbon sources. Along the axis of PC1 of the score plot (Figure 2A), the metabolite profiles in the lag phase were located on the positive side, while the metabolite profiles in the exponential and stationary phases were located on the negative side. In the loading plot (Figure 2B), most metabolites were distributed on the positive side of PC1. Therefore, by superimposing the scores and loading plots, it was revealed that the abundance of most intracellular metabolites were higher in the lag phase than in other phases. Along the axis of PC2, the metabolite profiles from the fucose media were located on the negative side, while the metabolite profiles from the glucose media were located on the positive side (Figure 2A). In the loading plot, most metabolites were evenly distributed along the axis of PC2 (Figure 2B). Therefore, the discrimination of metabolite profiles along the axis of PC2 suggest unique metabolite profiles based on sugars such as fucose and glucose.

**Figure 3:**
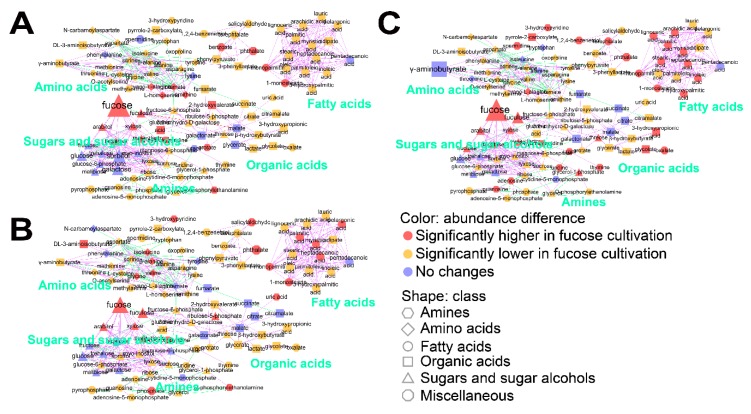
MetaMapp analysis of 102 intracellular metabolites of *E. coli* cultured on fucose and glucose as the carbon source in: (**A**) the lag phase; (**B**) exponential phase; and (**C**) stationary phase. Classes of metabolites are represented by shapes. Significant increases and decreases in metabolite abundance are represented by color (*p* < 0.05). Magnitudes of fold changes are represented by the size of symbols and labels. Biochemical and structural similarities are represented by the orange and gray edges, respectively (six replicates; three independent replicates × two technical replicates).

To understand the effect of fucose fermentation on cellular metabolism in more detail, intracellular metabolites of *E. coli* cultured in fucose and glucose media were compared in each growth phase using Student’s *t*-test (Table S4). Significantly changed metabolites (FDR adjusted *p*-values < 0.05) were visualized using MetaMapp (Figure 3). First, intracellular metabolites in the lag phase generated in fucose and glucose media were compared (Figure 3A). A total of 19 metabolites including intermediates of fucose fermentation, such as fucose and fuculose, and sugars indirectly connected with the pentose phosphate pathway, such as arabitol, xylose, and gluconic acid lactone, were at significantly higher levels during growth in fucose media (Figure 3A and Table S4). In contrast, a total of 16 metabolites, including intermediates of glycolysis and galactose metabolism such as glucose, glucose-6-phosphate, fructose, galactose, sorbitol, and myo-inositol, were significantly higher in glucose media (Figure 3A and Table S4). Second, intracellular metabolites in the exponential phase generated in fucose and glucose media were compared (Figure 3B). A total of 24 metabolites, including intermediates of fucose metabolism and six fatty acids, namely 1-monopalmitin, 1-monostearin, heptadecanoic acid, myristic acid, palmitic acid, and pelargonic acid, were significantly higher in fucose media (Figure 3B and Table S4). In contrast, a total of 19 metabolites, including intermediates of glycolysis and the TCA cycle such as succinate, malate, citrate, and fumarate, were significantly higher in glucose media (Figure 3B and Table S4). Third, intracellular metabolites in the stationary phase obtained in fucose and glucose media were compared (Figure 3C). A total of 35 metabolites, including intermediates of fucose metabolism and 10 fatty acids, namely 1-monopalmitin, 1-monostearin, 3-hydroxypalmitic acid, arachidic acid, heptadecanoic acid, lignoceric acid, linoleic acid, myristic acid, palmitic acid, and stearic acid, were significantly higher in fucose media (Figure 3C and Table S4). In contrast, a total of 16 metabolites, including intermediates of glycolysis and the TCA cycle, and γ-aminobutyrate, were significantly higher in glucose media (Figure 3C and Table S4). Taken together, in this study, intermediates of fucose metabolism, sugars indirectly connected with the pentose phosphate pathway, and intracellular fatty acids, were increased when grown on fucose, while intermediates of glycolysis and the TCA cycle, and γ-aminobutyrate, were elevated when grown on glucose.

### 2.4. Comparison of Intracellular and Extracellular Metabolite Profiles of E. coli on Fucose at Different Growth Phases

**Figure 4:**
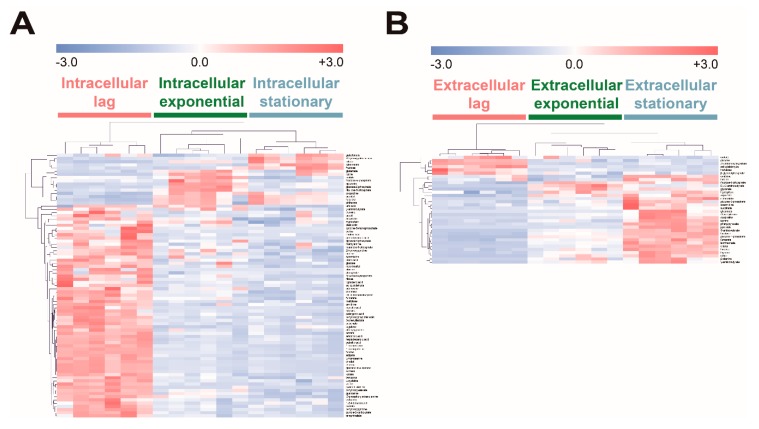
Hierarchical clustering analysis (FDR adjusted *p*-value < 0.01, ANOVA) of significantly increased or decreased (**A**) intracellular and (**B**) extracellular metabolites of *E. coli* in the lag, exponential, and stationary phases cultured on fucose as the carbon source. Clustering of the model was based on Pearson’s correlation coefficient and average linkage methods (six replicates; three independent replicates × two technical replicates).

To investigate the effect of fucose fermentation on intracellular metabolism, intracellular and extracellular metabolites of *E. coli* cultured in fucose media were compared at the three different growth phases, namely the lag, exponential, and stationary phases. To identify the significantly changed metabolites, ANOVA followed by post hoc Fisher’s LSD was performed. A total of 83 intracellular metabolites and 34 extracellular metabolites were significantly altered (Table S5; *p*-values < 0.05). The intensity of each significantly changed metabolite was converted to *z*-scores and then visualized using the HCA heat map based on Pearson’s correlation coefficient and average linkage methods (Figure 4). In the lag phase, most intracellular metabolites showed higher abundance (Figure 4A), while only a few extracellular metabolites, including pyrimidine synthesis pathway intermediates such as orotate and *N*-carbamoylaspartate, showed higher levels than in other growth phases (Figure 4B). In the exponential phase, intracellular metabolites related to gluconeogenesis and the pentose phosphate pathway, such as fructose-6-phosphate, glucose-6-phosphate, and ribulose-5-phosphate, exhibited higher abundance (Figure 4A), while four extracellular metabolites, fructose-6-phosphate, DL-3-aminobutyrate, glycerate, and tryptophan, showed higher abundance than in other growth phases (Figure 4B). In the stationary phase, intracellular organic acids, citrate, citramalate, and galactonate (Figure 4A), and most extracellular metabolites showed higher abundance than in other growth phases (Figure 4B). Intracellular and extracellular fucose showed the highest abundance in the lag phase, and gradually decreased as fucose was fermented (Figure 4). In contrast, intracellular and extracellular fuculose showed the lowest abundance in the lag phase, and gradually increased with fucose fermentation (Figure 4).

In summary, most intracellular metabolites showed higher abundance in the lag phase, and then tended to decrease, while most extracellular metabolites showed lower abundance in the lag phase, and tended to increase as fucose fermentation progressed. Interestingly, fuculose, which is an intermediate of fucose fermentation, showed the lowest abundance in the lag phase, and significantly increased as fucose fermentation progressed.

### 2.5. Comparison of Transcriptome and Cofactor Profiles of E. coli on Fucose and Glucose in the Exponential Phase

**Figure 5:**
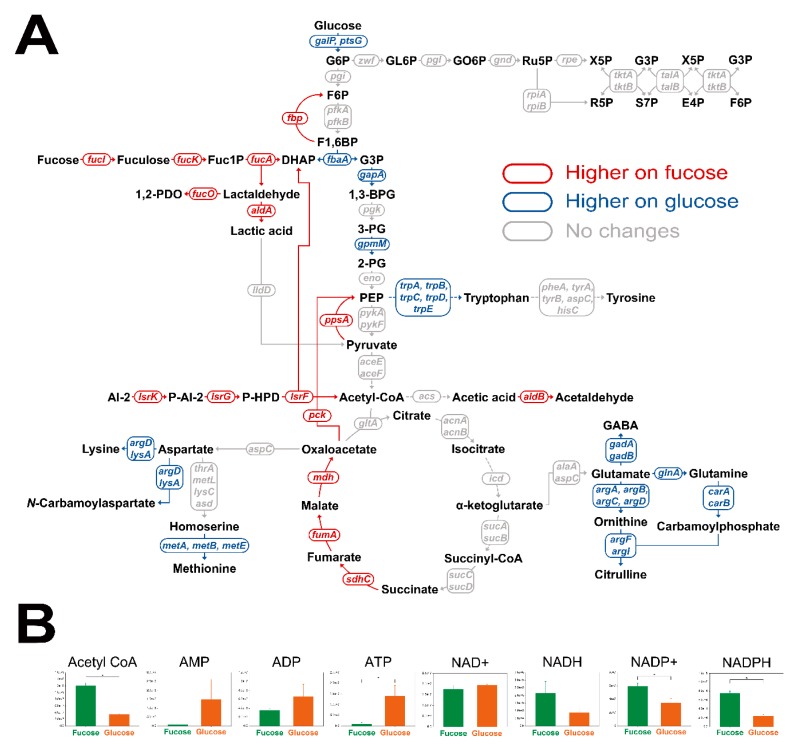
Comparison of transcription levels in (**A**) central carbon metabolism and (**B**) abundance of cofactors of *E. coli* in the exponential phase cultured on fucose and glucose. Significant changes to transcript levels are represented by color (*p*-value < 0.05, and fold changes > 2.0). Significant changes in cofactors are represented by * (three independent replicates; *p*-value < 0.05).

To investigate metabolic flows, transcriptomic and cofactor analyses were performed for *E. coli* cultured in fucose and glucose media in the exponential phase. Student’s *t*-test was performed to find significantly changed transcripts and cofactors. A total of 284 transcripts were found to be significantly different under two criteria (Table S2; *p*-value < 0.05 and fold change > 2.0). First, transcription levels in central carbon metabolism, including fucose fermentation, glycolysis, gluconeogenesis, the pentose phosphate pathway, and amino acid synthesis, were compared (Figure 5A). Transcripts involved in fucose fermentation, gluconeogenesis and the TCA cycle were up-regulated, while transcripts in glycolysis and amino acid synthesis were down-regulated in *E. coli* cultured on fucose, rather than on glucose. Transcripts involved in the pentose phosphate pathway showed no significant changes between the two culture conditions (Figure 5A). Second, cofactor metabolites, acetyl-CoA, AMP, ADP, ATP, NAD+, NADH, NADP+, and NADPH, were also compared (Figure 5B). Abundance of acetyl-CoA, NADP+, and NADPH were significantly higher, and ATP was significantly lower with fucose than with glucose (Figure 5B; *p*-value < 0.05).

**Figure 6:**
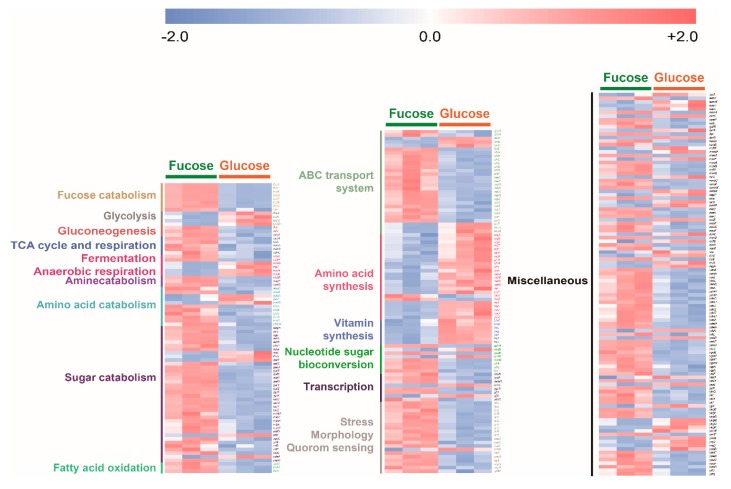
Heat map of 284 transcripts significantly changed (*p*-value < 0.05) in *E. coli* in the exponential phase cultured on fucose and glucose (three independent replicates).

To compare overall transcripts of *E. coli* cultured in fucose and glucose media, the expression level of each significantly changed transcript was converted into *z*-scores, classified into specific metabolic characteristics, and then visualized by using heat maps (Figure 6). Transcripts involved in fucose fermentation, gluconeogenesis, the TCA cycle and respiration, amine, amino acid and sugar catabolism, fatty acid oxidation, ABC transporter, nucleotide sugar bioconversion and stress, morphology, and quorum sensing were significantly higher in *E. coli* cultured in fucose media than in glucose media. Transcripts involved in glycolysis and amino acid and vitamin synthesis were significantly higher in *E. coli* cultured in glucose media than in *E. coli* cultured in fucose media (Figure 6).

## 3. Discussion

In this study, we found that the metabolic activity of *E. coli* responds differently when grown on two different carbon sources, fucose or glucose, as identified using metabolomics and transcriptomics. We also found that culturing *E. coli* on fucose leads to inefficient carbon metabolism, resulting in fuculose accumulation and slow growth. Such inefficient carbon metabolism may have been caused by ATP deficiency arising from gluconeogenesis and quorum sensing. To more efficiently convert fucose into bio-based products by engineered *E. coli* at higher yields and productivities, fuculose catabolism, gluconeogenesis, and quorum sensing pathways need to be manipulated by metabolic engineering. The raw metabolome and transcriptome data have been uploaded to provide useful information for other researchers.

Our metabolomic and transcriptomic analyses suggested that severe depletion of ATP is mainly caused by up-regulation of gluconeogenesis in *E. coli* cultured on fucose. Of central metabolic activities, fucose fermentation, gluconeogenesis, and the TCA cycle, were up-regulated, while glycolysis was down-regulated with growth on fucose, compared to glucose (Figure 5A). In fucose media, the fucose catabolic gene expression (Figure 5A) and intermediate metabolites (Figure 3) were significantly higher when fucose was supplied as the carbon source [20]. In contrast, in glucose media, glycolytic gene expression (Figure 5A) and intermediate metabolites (Figure 3) were significantly higher when glucose was the carbon source [28]. Interestingly, gluconeogenic genes, such as *fbp*, *ppsA*, and *pck,* were significantly up-regulated in fucose media (Figure 5A). The abundance of fructose-1,6-bisphosphate (FBP), which is an intermediate of upper glycolysis, is a key regulator of gluconeogenesis [29]. The upper glycolysis intermediates such as FBP are almost absent in *E. coli* on fucose since these metabolites are synthesized via gluconeogenesis [21,29]. The lack of FBP induces the up-regulation of *cra* (Figure 6), which up-regulates gluconeogenetic genes such as *fbp*, *pck*, and *ppsA*, and TCA cycle genes such as *mdh*, *fumA*, and *sdhC* (Figure 5A) [29]. As a result, cells grown on fucose synthesize upper glycolysis intermediates via gluconeogenesis [21,29]. However, gluconeogenesis is an expensive process, using large amounts of ATP and DHAP [21,29], resulting in a lack of ATP in *E. coli* grown on fucose (Figure 5B).

Up-regulated quorum sensing metabolism causes ATP deficiency and accumulation of acetyl-CoA in *E. coli* on fucose. Uptake of fucose is known to up-regulate quorum sensing genes in gut microorganisms such as *Roseburia inulinivorans* but its mechanism is not known yet [30,31]. Likewise, *E. coli* cultured on fucose showed up-regulation of quorum sensing genes such as *lsrA*, *lsrB*, *lsrC*, *lsrD*, *lsrF*, *lsrG*, and *lsrK* (Figure 6). The up-regulated genes such as *lsrA*, *lsrC*, and *lsrD* induce uptake of *S*-4,5-dihydroxy-2,3-pentanedione (autoinducer-2, AI-2) [32]. Then, the protein *lsrK* phosphorylates AI-2 to phospho-*S*-4,5-dihydroxy-2,3-pentanedione (P-AI-2), consuming ATP (Figure 5A) [32]. The protein product of the *lsrG* gene converts P-AI-2 into hydroxy-2,4-pentadione-5-phosphate (P-HPD) (Figure 5A) [32]. The protein encoded by the last gene, *lsrF,* converts P-HPD into DHAP and acetyl-CoA using CoA-SH (Figure 5A) [32]. Acetyl-CoA is a key intermediate metabolite for ATP synthesis through two main pathways, the TCA cycle and acetic acid production [33]. However, the amount of acetyl-CoA that enters these pathways is limited, and thus the expression levels of genes related to these pathways are the same for growth in both fucose and glucose media (Figure 5A) [33]. Thus, the acetyl-CoA introduced from AI-2 accumulates, rather than being used for energy generation (Figure 5B). Since the synthesis and degradation of AI-2 consumes ATP [32,34], *E. coli* on fucose undergoes ATP deficiency much more severely than that on glucose (Figure 5B). Similarly, *Sulfolobus solfataricus* grown on fucose showed significantly higher levels of intracellular CoA metabolites than those grown on glucose [21].

The accumulation and export of fuculose, probably caused by ATP deficiency and insufficient expression of the *fucK* gene, may result in large amounts of wasted carbon sources, as well as an inefficient carbon flux in *E. coli* on fucose. In fucose fermentation, the protein of *fucI* isomerizes fucose into fuculose, which is then phosphorylated to fuculose-1-phosphate by the protein *fucK* using ATP (Figure 5A) [21]. As mentioned above, *E. coli* cultured on fucose undergoes severe ATP depletion due to active gluconeogenesis and quorum sensing (Figure 5B). Due to the deficiency of ATP, the metabolic activity of the *fucK* protein is not enough to phosphorylate all the available fuculose (Figure 5A). Instead, the remaining intracellular fuculose accumulates (Figure 4A) and may be exported extracellularly (Figure 4B). If fuculose could be catabolized, rather than accumulating, the productivity and yield of fucose into bio-based products would be significantly increased.

Metabolic engineering can be exploited to increase the productivity and yield of converting fucose into bio-based products by solving the inefficient carbon flux and reducing wasted carbon sources, as observed in this study [1,3,22]. In fucose fermentation in this study, it was found that the slow growth rate and yield may be caused by ATP deficiency (Figure 5B) and export of fuculose (Figure 4B). Metabolic engineering for reducing the expense of ATP caused by unnecessary metabolism can increase productivity and yields [1,3,22]. For example, the down-regulation of the gluconeogenetic and quorum sensing genes, *pck*, *ppsA*, and *lsrK*, may preserve large amounts of ATP, while the up-regulation of fucose catabolic genes, such as *fucK* and *fucA,* can increase the fucose consumption rate and its product yield. Modifying expression levels of such genes may increase the productivity and yield by facilitating conversion of fuculose and acetyl-CoA into bio-based products in metabolically engineered *E. coli*.

In this study, overall changes to metabolic activities in *E. coli* utilizing fucose were investigated by a multi-omics approach composed of metabolomic and transcriptomic analyses. Our findings indicate that cells utilizing fucose undergo inefficient carbon fluxes caused by the accumulation of acetyl-CoA and fuculose. These phenomena are caused by ATP deficiency due to gluconeogenesis and quorum sensing, and eventually result in slow growth and production rates of *E. coli* on fucose. The metabolic bottlenecks suggested here can be resolved by using metabolic engineering to design microbial strains for producing bio-based products from fucose more efficiently. To the best of our knowledge, this is the first multi-omics study of metabolism in *E. coli* cultured on fucose.

## 4. Materials and Methods

### 4.1. Strains, Media, and Culture Conditions

*E. coli* K12 MG1655 was cultured in 100 mL of medium in 250 mL flasks at 37 °C and 200 rpm under aerobic conditions, with three independent replicates. The culture conditions included M9 medium containing 1% L-fucose (*w*/*v*) or 1% glucose (*w*/*v*), and the initial pH of each medium was 6.8 ± 0.2. Samples for metabolome analysis were collected during the lag, exponential, and stationary phases (six replicates; three independent replicates × two technical replicates). Samples for transcriptome and cofactor analyses were collected during the exponential phase (three independent replicates).

### 4.2. Preparation of Intracellular and Extracellular Metabolite Samples

Intracellular metabolites of *E. coli* K12 MG1655 were extracted using the fast filtration method with slight modifications [35]. Cells cultured in M9 fucose or glucose media were obtained by filtration of 1 mL of cell culture using a nylon membrane filter (0.20 µm pore size, Whatman, Piscataway, NJ, USA) under vacuum. Then, the cell mass on the membrane filter was washed with 5 mL of distilled water. A mixture of cell mass and membrane filter were immediately mixed with 10 mL of acetonitrile/water (1:1, *v*/*v*) extraction solvent at −20 °C. Then, the mixture was immersed in liquid nitrogen to stop cellular metabolism and any further changes in metabolite levels. The entire metabolite preparation procedures were completed within 30 s. The extraction mixture was thawed on ice, sonicated for 5 min, vortexed for 3 min, and centrifuged at 16,100× *g* for 10 min at 4 °C. The supernatant containing intracellular metabolites was collected and vacuum dried at room temperature.

To separate extracellular metabolites of *E. coli* K12 MG1655, 1 mL of the cell culture was centrifuged at 16,100× *g* for 10 min at 4 °C. A 10 µL aliquot of the supernatant containing extracellular metabolites was collected and vacuum dried.

### 4.3. GC/TOF–MS and HPLC Analyses of Intracellular and Extracellular Metabolites

For the qualification and quantification of intracellular and extracellular metabolites using GC/TOF–MS, two derivatization methods, methoximation and silylation, were performed on metabolite samples. For methoximation, 10 µL of 40 mg/mL methoxyamine hydrochloride in pyridine (Sigma-Aldrich, St. Louis, MO, USA) was added to the metabolite samples, and the mixture was incubated at 30 °C for 90 min. For silylation, 45 µL of *N*-methyl-*N*-trimethylsilyl-trifluoroacetamide (Fluka, Buchs, Switzerland) was added to the mixture, which was incubated at 37 °C for 30 min. As retention index markers, a mixture of fatty acid methyl esters, including methyl forms of C8, C9, C10, C12, C14, C16, C18, C20, C22, C24, C26, C28, and C30 fatty acids, was added to the derivatized sample.

An Agilent 7890B GC (Agilent Technologies, Santa Clara, CA, USA) platform coupled with a Pegasus HT–TOF MS system (LECO, St. Joseph, MI, USA) was used for analysis of intracellular metabolites. A 0.5 µL aliquot of the derivatized sample was injected into the GC, in splitless mode. The metabolites were separated on an RTX-5Sil MS column (30 m length, 0.25 mm inner diameter, and 0.25 µm film thickness; Restek, Bellefonte, PA, USA) with an additional 10 m guard column. The oven temperature was initially set at 50 °C for 1 min, and was then ramped to 330 °C at a rate of 20 °C/min, and held at 330 °C for 5 min. Mass spectra were recorded over a mass range of 85–500 *m/z* at an acquisition rate of 10 spectra/s. The temperatures of the ion source and transfer line of the TOF–MS were set at 250 °C and 280 °C, respectively. The sample was ionized by electron impact at 70 eV.

For the identification and quantification of extracellular metabolites such as 1,2-PDO, acetic acid, fucose, and glucose, an HPLC system equipped with a refractive index detector, Agilent 1100 (Agilent Technologies, Santa Clara, CA, USA), and an Aminex HPX-87H organic acid column (Bio-Rad, Hercules, CA, USA) was used. The 0.01 N H_2_SO_4_ was eluted at a constant flow rate of 0.5 mL/min at 65 °C as the mobile phase.

### 4.4. GC/TOF–MS Data Processing and Statistical Analyses

Chroma TOF Software C version (LECO, St. Joseph, MI, USA) was used for automated detection of peaks and deconvolution of mass spectra. These pre-processed data were further processed using BinBase (http://binbase.sourceforge.net) [36] for the identification of metabolites by matching mass spectra and retention indices of peaks with the customized reference mass spectral and retention index libraries that were acquired using authentic standards under identical data acquisition parameters of the Fiehn and NIST libraries [36,37]. Peaks showing mass spectral similarities above 700 in comparison to their authentic standards were regarded as identical to the authentic standards. Intensities of the identified metabolites were reported as peak heights of their unique ion intensities. To treat missing values, the lowest background intensity was subtracted from the intensity of the quantified ion in its retention time region ±5 s using MZmine software (2.0; http://mzmine.sourceforge.net) [36]. The intensities of metabolites were normalized by using the cellular dry weight of each sample. The normalized data were then used in multivariate and univariate statistical analyses, namely, PCA, hierarchical clustering analysis (HCA), student’s *t*-test, and analysis of variance (ANOVA), followed by Fisher’s least significant difference (LSD) and MetaMapp analysis [38]. PCA was performed using SIMCA-P+ software (Version 12.0; Umetrics AB, Umea, Sweden). Hierarchical clustering analysis was performed by using MultiExperiment Viewer application [39]. Student’s *t*-test and ANOVA were performed by using Metaboanalyst [40]. The metabolome data analyzed by GC/TOF–MS have been uploaded (Table S1A,B).

### 4.5. Quality Controls for GC/TOF–MS Analysis

To maintain high-quality data in metabolite analysis, daily quality control was performed. Two method blanks, involving all reagents and equipment used to control laboratory contamination, and four calibration curve samples composed of 31 pure reference compounds were analyzed under the same analysis protocol [41]. Intervention limits were established based on standard operating procedures to ensure basic validation of the instrument for metabolite profiling [41].

### 4.6. LC/MS Analysis of Energy and CoA Metabolites

For analysis of cofactor metabolites, the dried metabolite extracts were resuspended in 200 μL water. Ultimate 3000 UHPLC (Thermo Fisher Scientific, Waltham, MA, USA) and Q-Exactive MS (Thermo Fisher Scientific, Waltham, MA, USA) with a heated electrospray ionization source were used. A 10 μL aliquot of sample was injected and separated by an ACQUITY UPLC HSS T3 column (150 mm × 2.1 mm × 1.8 μm; Waters, Milford, MA, USA), and the column was maintained at 35 °C. The mobile phase A consisted of water containing 10 mM tributylamine and 15 mM acetic acid, and mobile phase B consisted of pure methanol. The gradient of the mobile phase was programmed at 0 min with 97% A and 3% B, and at 15 min with 25% A and 75% B, and the flow rate of the mobile phase was 0.25 mL/min. Negative spray voltage was 3.0 kV, and sheath gas flow rate was 40 mL/min, auxiliary gas flow rate was 10 mL/min, auxiliary gas heater temperature was 350 °C, and capillary temperature was 325 °C. The resolution was 17,500 full width at half maximum (FWHM), and mass extraction window was 5 ppm. The maximum injection time was set at 200 s, a scan range of *m/z* was 300−1000, and the mass inclusion list was used for targeted selected ion monitoring (SIM) analysis, and the expected retention times of target analytes with a 2 min time window. The metabolome data analyzed by LC/MS have been uploaded (Table S1C).

### 4.7. RNA Isolation and RNA Library Construction

To construct cDNA libraries with a TruSeq RNA library kit (Illumina, San Diego, CA, USA), 1 µg of total RNA was used. mRNA was purified from total RNA by polyA selection, and then chemically fragmented and converted into single-stranded cDNA with random hexamer priming. A second cDNA strand was generated to create double-stranded (ds) cDNA for construction of the TruSeq library. The short ds cDNA fragments were then connected using sequencing adapters, and suitable fragments were separated by agarose gel electrophoresis. RNA libraries were generated by PCR amplification and quantified by qPCR, in accordance with qPCR Quantification Protocol Guide (Illumina, San Diego, CA, USA), then qualified using an Agilent 2100 Bioanalyzer instrument (Agilent Technologies, Santa Clara, CA, USA).

### 4.8. Statistical Analysis of Gene Expression Levels

The cDNA library was sequenced using the HiSeq 2000 platform (Illumina, San Diego, CA, USA), and sequence analysis was performed by Macrogen, Inc. (Seoul, Korea). Validation of sequence quality was performed using FastQC (Version 0.10.0; Babraham Institute, Babraham, UK).

To compare gene expression profiles of *E. coli* cultured in either fucose or glucose media, their sequences were aligned to the cDNA of *E. coli* K12 MG1655 using Bowtie Version 1.1.2 (http://bowtie.cbcb.umd.edu) [42]. Read counts, represented as expression abundance of each gene in each sample, were extracted from the alignment file using HTSeq [43]. To calculate gene expression levels, the reads per kilobase per million mapped reads (RPKM) method was used. The RPKM values of filtered transcripts were transformed by log 2 and added to 1, and the data were then normalized using the quantile normalization method. The transcriptome data have been uploaded (Table S2).

## Supplementary Materials

The following are available online at http://www.mdpi.com/1660-3397/17/2/82/s1, **Table S1**: (**A**) Intracellular metabolome data analyzed by GC/TOF–MS; (**B**) Extracellular metabolome data analyzed by GC/TOF–MS; (**C**) Intracellular cofactor metabolites analyzed by LC–MS. **Table S2**: Identification of 4414 transcripts (mRNA). **Table S3**: Loading values of 102 intracellular metabolites in the PCA model. **Table S4**: Student’s t-test for the comparison of intracellular metabolites of *E. coli* cultivated in fucose and glucose media. **Table S5**: (**A**) Analysis of variance test for the comparison of 102 intracellular metabolites of *E. coli* at different growth phases: early, exponential, and stationary phases; (**B**) Analysis of variance test for the comparison of 53 extracellular metabolites of *E. coli* at different growth phases: early, exponential, and stationary phases.
